# Anti-Inflammatory Activity of Cyanobacterial Serine Protease Inhibitors Aeruginosin 828A and Cyanopeptolin 1020 in Human Hepatoma Cell Line Huh7 and Effects in Zebrafish (*Danio rerio*)

**DOI:** 10.3390/toxins8070219

**Published:** 2016-07-14

**Authors:** Susanne Faltermann, Simon Hutter, Verena Christen, Timm Hettich, Karl Fent

**Affiliations:** 1 School of Life Sciences, University of Applied Sciences and Arts Northwestern Switzerland (FHNW), Gründenstrasse 40, CH-4132 Muttenz, Switzerland; susanne.faltermann@fhnw.ch (S.F.); simon.hutter@students.fhnw.ch (S.H.); verena.christen@fhnw.ch (V.C.); timm.hettich@fhnw.ch (T.H.); 2 Department of Environmental Systems Science, Institute of Biogeochemistry and Pollution Dynamics, Swiss Federal Institute of Technology (ETHZ), CH-8092 Zürich, Switzerland

**Keywords:** aeruginosin, cyanopeptolin, microcystin, anti-inflammatory, human hepatoma cells, zebrafish, transcription analysis, cytochrome P450

## Abstract

Intensive growth of cyanobacteria in freshwater promoted by eutrophication can lead to release of toxic secondary metabolites that may harm aquatic organisms and humans. The serine protease inhibitor aeruginosin 828A was isolated from a microcystin-deficient *Planktothrix* strain. We assessed potential molecular effects of aeruginosin 828A in comparison to another cyanobacterial serine protease inhibitor, cyanopeptolin 1020, in human hepatoma cell line Huh7, in zebrafish embryos and liver organ cultures. Aeruginosin 828A and cyanopeptolin 1020 promoted anti-inflammatory activity, as indicated by transcriptional down-regulation of interleukin 8 and tumor necrosis factor α in stimulated cells at concentrations of 50 and 100 µmol·L^−1^ aeruginosin 828A, and 100 µmol·L^−1^ cyanopeptolin 1020. Aeruginosin 828A induced the expression of *CYP1A* in Huh7 cells but did not affect enzyme activity. Furthermore, hatched zebrafish embryos and zebrafish liver organ cultures were exposed to aeruginosin 828A. The transcriptional responses were compared to those of cyanopeptolin 1020 and microcystin-LR. Aeruginosin 828A had only minimal effects on endoplasmic reticulum stress. In comparison to cyanopeptolin 1020 our data indicate that transcriptional effects of aeruginosin 828A in zebrafish are very minor. The data further demonstrate that pathways that are influenced by microcystin-LR are not affected by aeruginosin 828A.

## 1. Introduction

Cyanobacteria (formerly called blue green algae) have inhabited our planet for over 3.5 billion years and produce oxygen by photosynthesis. Compared to green algae, cyanobacteria possess distinct advantages, such as fixation of nitrogen and gas vesicles which allow vertical movements. They produce numerous secondary metabolites, which have many different functions including growth inhibition of competing phytoplankton species [1], increase of iron availability [2], and defense against parasites [3]. Some crude extracts of cyanobacteria showed estrogenic activity but so far no specific compounds could be attributed to this activity [4]. Cyanobacteria may also produce toxic secondary metabolites. The cyclic peptides of the microcystin (MC) family, for example, may be involved in hepatocancerogenic activity in humans [5]. Various molecular effects, including induction of endoplasmic reticulum (ER) stress, have been demonstrated in human hepatoma cells [6], and zebrafish [7]. Although humans and a variety of organisms may be negatively affected, the major targets are grazers [8,9], including zooplankton (daphnids) that are sensitive to MC [10].

Not all cyanobacteria are able to produce MCs [11]. A gene cluster coding for the enzyme complex that synthesizes the peptides is needed for their synthesis [12]. Point mutations within this cluster can result in the loss of MC production. Nevertheless, even MC deficient strains such as *Planktothrix rubescens* strain 91/1, can successfully defend against grazers. Recently, a sulfate and chlorine containing linear molecule of the aeruginosin family, aeruginosin 828A (AG 828A), was isolated and demonstrated to be toxic to *Thamnocephalus platyurus* with an LC_50_ of 22.4 µmol·L^−1^ [11]. Members of the aeruginosin family contain a common structural element, 2-carboxy-6-hydroxyoctahydroindol, named Choi [13]. They exhibit serine protease inhibitory activity. Aeruginosin variants have been demonstrated to inhibit proteases from the human blood coagulation cascade such as thrombin [14]. Aeruginosin 865 was further shown to have anti-inflammatory activity [15]. Serine protease inhibitors also affect digestive enzymes such as trypsin [16], and they may negatively interfere with the growth of grazers [17,18].

Aeruginosins belong to cyanobacterial serine protease inhibitors along with cyanopeptolins, microviridins and anabaenapeptins [13]. Cyanopeptolins are characterized by 3-amino-6-hydroxy-2-piperidone (Ahp) [19] as their common structural element. A member of this family, cyanopeptolin 1020 (CP 1020), was recently isolated, and shown to be toxic to *Thamnocephalus platyurus* [16]. In contrast to AG 828A, CP 1020 is produced by a MC-synthesizing *Microcystis* strain. CP 1020 was found to influence the transcription of genes involved in circadian rhythm and DNA damage repair in zebrafish embryos [20]. In contrast to MCs and CP 1020, molecular effects and modes of actions of aeruginosins are largely unknown, particularly in human cells and fish. 

Recently, AG 828A was hypothesized to compensate functionally for the loss of MC toxicity [11]. The basis of this hypothesis was that the loss of MC production in *Planktothrix rubescens*, which may result from a point mutation within the synthesis gene cluster for example, seems to have no disadvantage for the cyanobacterial strain [11]. The LC_50_ value of AG 828A for *Thamnocephalus platyurus* was in the range of that of MC [21]. However, the modes of actions differ. While MCs inhibit protein phosphatases [22], AG 828A inhibits protein proteases [11,14]. 

Until now, cellular uptake mechanisms by which aeruginosins enter cells are unknown. Recently, we have shown that MC-LR utilizes organic anion transporting polypeptides for cellular uptake in zebrafish [23]. Moreover, we showed that the human hepatoma cell line Huh7 is a suitable model for studying molecular effects of cyanobacterial hepatotoxins, which contrasts with assessments in a zebrafish liver cell line that did not sufficiently express uptake transporters and was thus insensitive to them [6,23]. Therefore, we employed Huh7 cells to investigate molecular effects of AG 828A.

In addition, we wanted to evaluate potential molecular effects in fish in comparison to CP 1020 and MC-LR by transcription analysis in the liver of zebrafish embryos and adults. Our concept was to focus on target genes that were previously demonstrated to be affected by CP 1020 [20]. Although AG 828A and CP 1020 are non-ribosomal oligopeptides that share serine protease inhibiting activity [11,16], they may differ in their effects. To this end, we evaluated the expression of the same target genes known to be altered by CP1020. Furthermore, we aimed to investigate the similarity or dissimilarity of effects of AG 828A to MC-LR. As AG 828A may compensate for the loss of MC in *Planktothrix* [11], we also evaluated additional genes that were affected by MC-LR [6]. 

## 2. Results

### 2.1. Effects of Aeruginosin 828A in Human Hepatoma Cell Line Huh7

Huh7 cells functionally express uptake transporters; as molecular effects of MC-LR have previously shown in this suitable cell line, we first focused on potential effects of AG 828A and compared them to CP 1020.

#### 2.1.1. Anti-Inflammatory Effects of AG 828A and CP 1020

**Figure 1:**
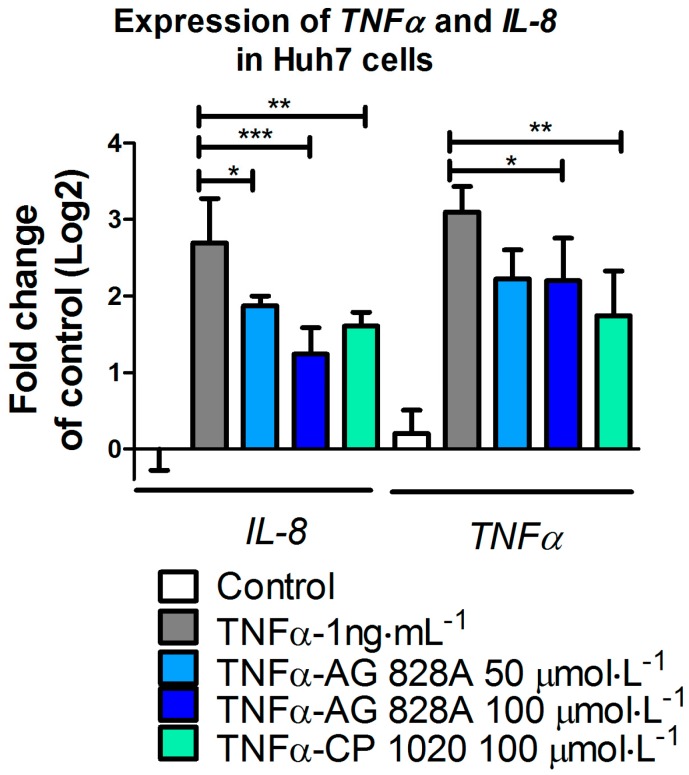
Alteration of *IL8* and tumor necrosis factor alpha (*TNFα*) transcripts (mRNA) of genes involved in inflammation in Huh7 cells stimulated with TNFα. Exposure to 50 and 100 µmol·L^−1^ AG 828A and 100 µmol·L^−1^ CP 1020 prior to TNFα treatment. Significant changes in transcript levels compared to TNFα exposure only are indicated by asterisks (* *p* < 0.05; ** *p* < 0.001; *** *p* < 0.0001).

Treatment with the cytokine tumor necrosis factor alpha (TNFα) led to transcriptional up-regulation of several marker genes including *interleukin8* (*IL8*) and *tumor necrosis factor α* (*TNFα*) itself (Figure 1). Incubation with TNFα for 24 h results in a strong increase of *IL8* and *TNFα* transcription. In contrast, pre-exposure to the protease inhibitor AG 828A significantly lowered the effect of TNFα in Huh7 cells. The reduction in transcriptional up-regulation of *IL8* and *TNFα* is significant for both genes after exposure to AG 828A. The effect on *IL8* transcription was more prominent. Similar effects were observed with CP 1020 at a concentration of 100 µmol·L^−1^.

#### 2.1.2. CYP1A Induction by AG 828A

**Figure 2:**
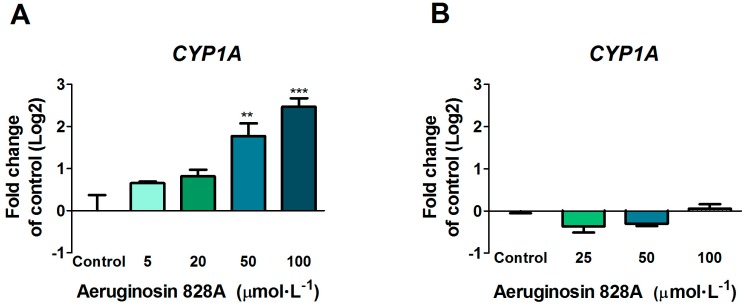
Transcripts of *CYP1A* after exposure of Huh7 cells to different concentrations of AG 828A for 24 h. (**A**) in Hank’s Balanced Salt Solution (HBSS) media; (**B**) in culture media without fetal bovine serum (FBS). (** *p* < 0.001; *** *p* < 0.0001).

Exposure to different concentrations of AG 828A in Hank’s Balanced Salt Solution (HBSS buffer) resulted in a significant, concentration-dependent increase in *CYP1A* transcription (Figure 2A). Expression was slightly up-regulated at 5 µmol·L^−1^ and significantly increased at 25, 50, and 100 µmol·L^−1^. At the highest concentration of 100 µmol·L^−1^, *CYP1A* expression was increased with a fold change (log2) of 2.47.

In contrast, exposure to AG 828A in cell culture media without fetal bovine serum (FBS) did not induce changes in *CYP1A* transcripts (Figure 2B). In addition, we analyzed transcriptional expression of genes that are, like *CYP1A*, regulated by the aryl hydrocarbon receptor (AHR); these included *glutathione-S-transferase a1* (*GSTa1*), *uridine diphospho-glucuronosyltransferase 1A6* (*UGT1A6*), ornithine-decarboxylase (*ODC*) and *nicotinamid adenindinucleotide phosphate* (*NADPH*) *chinon oxidoreductase* (*NQO*). However, no changes in transcriptional expression were found (Figure S1).

**Figure 3:**
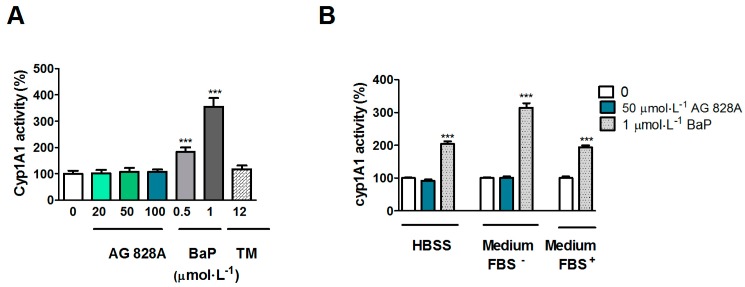
CYP1A enzyme activity (ethoxyresorufin-O-deethylase (EROD) activity) in Huh7 cells after exposure to AG 828A, positive control for CYP1A, benzo[*a*]pyrene (BaP), and endoplasmic reticulum (ER) stress, tunicamycin (TM), for 24 h. (**A**) Exposure in culture medium containing fetal bovine serum (FBS); (**B**) Exposure of Huh7 cells to AG 828A in HBSS and culture media with or without FBS. Values are presented as mean ±SD. Significant changes in CYP1A activity compared to control (0) are indicated by asterisks (*** *p* < 0.0001).

To analyze for CYP1A enzyme activity, ethoxyresorufin-*O*-deethylase (EROD) activity was assessed after exposure of Huh7 cells to AG 828A in cell culture medium supplemented with FBS. While the positive control, benzo[*a*]pyrene (BaP) in concentrations of 0.1 and 1 µmol·L^−1^, led to up-regulation, no increase in EROD activity was observed after exposure of Huh7 cells for 24 h to concentrations of 20, 50, and 100 µmol·L^−1^ AG 828A (Figure 3A). The second positive control for EROD induction, 12 µmol·L^−1^ tunicamycin [23], did not induce EROD activity. In contrast, BaP significantly induced EROD activity in the present study. The induction was independent from the experimental setup. However, in Huh7 cells exposed to AG 828A in HBSS and cell culture media without FBS, no induction of EROD activity occurred.

#### 2.1.3. Transcriptional Alteration of Genes Belonging to Different Toxicologically Relevant Pathways

We previously demonstrated that the non-ribosomal peptide MC-LR led to induction of ER stress in Huh7 cells [6]. As aeruginosins may compensate for MC-deficiency in cyanobacteria [11], the question arises whether AG 828A affects similar general toxicity pathways such as MC-LR, even though the key mode of action differs. Consequently, we analyzed whether AG 828A alters transcription of genes involved in ER stress or other potential toxicologically relevant pathways in Huh7 cells. To this end we evaluated the transcription of genes known to be influenced by microcystin in Huh7 cells exposed to AG 828A in HBSS buffer. The aim was to evaluate the similarity/dissimilarity of these non-ribosomal peptides. The genes included the *binding immunoglobulin protein* (*BIP*), *CCAAT/enhancer binding protein* (C/EBP) *homologous protein* (*CHOP*), *spliced X-box binding protein 1* (XBP-1s), *growth arrest and DNA damage-inducible protein 34* (*GADD34*), *death receptor 5* (*DR5*), *jun proto-oncogene* (*CJUN*), *fos proto-oncogene* (*CFOS*), and *caspase 8* (*CASP8*). The data in Figures S2 and S3 show that a significant induction was found for the ER stress marker gene, *BIP*, only. For all other genes belonging to different pathways (Table 1), including inflammation and urokinase activation system, no significant changes in transcriptional expression were found (Figure S3).

### 2.2. Effects of Aeruginosin 828A in Zebrafish Exposed to AG 828A

To identify potential molecular effects, transcription of selected target genes was analyzed in zebrafish eleuthero-embryos after exposure to AG 828A in comparison to controls. The target genes were selected as follows. First, due to the same modes of action of AG 828A and CP1020 that act as protease inhibitors, genes altered by CP1020 were evaluated after exposure of zebrafish to AG 828A. The selected genes were derived from our global transcription analysis in zebrafish eleuthero-embryos [20]. Second, genes indicative of a more generalized toxicological effect, including oxidative stress,AHR pathway, inflammation, mitogen-activated protein kinase (MAPK) signaling, hormone signaling, apoptosis and others were investigated (Table 1). These toxicological targets were evaluated to obtain a more complete pattern of potential expressional responses relevant for the toxicological action. Third, ER stress was investigated for comparison with MC-LR, as this cyanotoxin was shown to induce this effect [6]. Moreover, we wanted to test whether AG 828A compensates for toxicity of MC-LR. The targeted genes and pathways are indicated in Table 1.

#### 2.2.1. Transcriptional Effects on Diverse Toxicological Pathways

**Figure 4:**
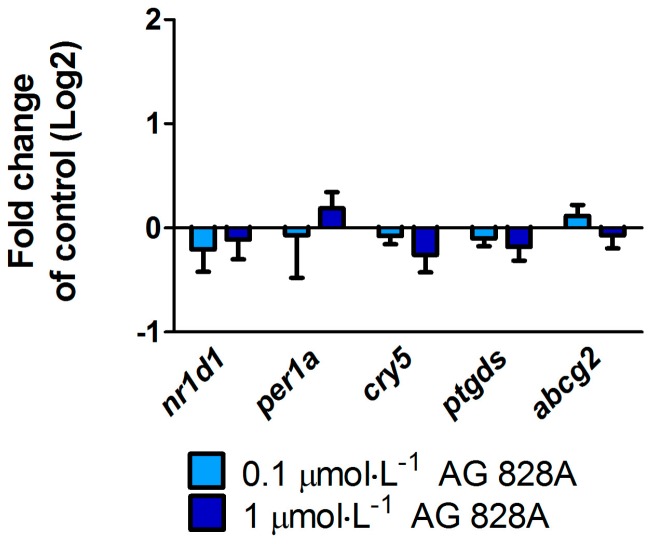
Abundance of transcripts (mRNA) of genes related to circadian rhythm (*nuclear receptor subfamily 1*, *group D*, *member 1* (*nr1d1*) or *period 1* (*per1*), DNA damage repair (cryptochrome 5 (*cry5*)), prostaglandin synthetase (*prostaglandin D2 synthase* (*ptgds*)) and efflux transporter (*adenosine triphosphate-*(*ATP*) *binding cassette transporter sub-family G member 2* (*abcg2*)) following exposure to 0.1 and 1 µmol·L^−1^ AG 828A from 3 to 7 dpf in zebrafish eleuthero-embryos.

Exposure of zebrafish embryos to 0.1 and 1 µmol·L^−1^ AG 828A started after hatching, three days post fertilization (dpf), and lasted until seven dpf. The abundance of transcripts of selected genes related to circadian rhythm, such as *nuclear receptor subfamily 1*, *group D*, *member 1* (*nr1d1*) and *period 1* (*per1*), or DNA damage repair, *cryptochrome 5* (*cry5*), did not change (Figure 4). Furthermore, no transcriptional changes were found *for prostaglandin D2 synthase* (*ptgds*) or the efflux transporter *adenosine triphosphate-*(*ATP*) *binding cassette transporter sub-family G member 2* (*abcg2*).

To evaluate additional toxicological pathways that may be affected by AG 828A, we analyzed the transcription of genes involved in cellular stress responses, detoxification, and MAPK signaling, as well as AHR regulated genes after exposure of zebrafish eleuthero-embryos to AG 828A. Furthermore, alteration of these gene transcripts was assessed in zebrafish liver organ cultures. However, no significant alterations of gene expression were found (Figures S4–S6, Figures S7–S11). These data strongly suggest that AG 828A bears no general toxicologically relevant effect pattern besides the anti-inflammatory action and *CYP1A* induction.

#### 2.2.2. Transcriptional Effects on Genes Involved in ER Stress and Estrogenic Response in Zebrafish Eleuthero-Embryos and in Liver Organ Cultures

**Figure 5:**
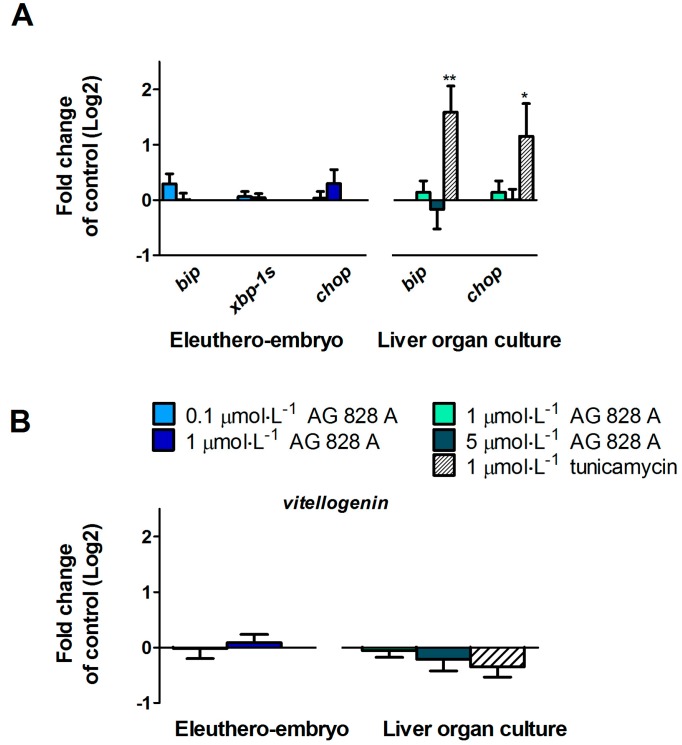
Alteration transcripts (mRNA) of genes in zebrafish eleuthero-embryos and liver organ culture, following exposure to different concentrations of AG 828A. (**A**) Selected target genes involved in ER stress; (**B**) Transcriptional expression of estrogenic marker gene vitellogenin. Significant changes compared to control are indicated by asterisks (* *p* < 0.05; ** *p* < 0.001).

As the aeruginosin variant 828A is produced by an MC-deficient *Planktothrix* strain, it was suggested that this toxin may compensate for the lack of toxic MCs [11]. We previously demonstrated that MC-LR led to induction of ER stress in zebrafish liver organ cultures, but not in zebrafish eleuthero-embryos [7]. To test the hypothesis whether AG 828A compensates for toxicity of MC-LR, we investigated the action of AG 828A on ER stress. To this end, marker genes for ER stress, including *bip*, *xbp-1s*, and *chop* as a marker for ER stress induced apoptosis, were investigated. No alterations of transcriptional expression of *bip* and *chop* occurred in zebrafish eleuthero-embryos exposed to 0.1 and 1 µmol·L^−1^ AG 828A for 4 days (Figure 5A). Similarly, no induction of any marker gene was found after exposure of zebrafish liver organ cultures to 1 and 5 µmol·L^−1^ AG 828A for 5 h (Figure 5A).

Induction of yolk precursor protein *vitellogenin* (*vtg*) represents a marker for estrogenic activity of compounds in fish. Microcystin-containing crude extracts were shown to induce *vtg* expression in zebrafish eleuthero-embryos [4]. Moreover, it was demonstrated that this effect is not induced by MC but rather by unidentified compounds. Therefore, the reason for the estrogenic activity is unknown. To test whether or not AG 828A may contribute to the estrogenic activity of such an extract, we determined the expression of the vitellogenin transcript after exposure of eleuthero-embryos and liver organ cultures to AG 828A. Figure 5B shows that AG 828A did not affect the expression of vitellogenin.

## 3. Discussion

The protease inhibitor AG 828A is produced by a microcystin-deficient *Planktothrix* strain. We evaluated the activity of this aeruginosin with regard to a series of important, toxicologically-relevant, biological pathways in order to understand obtain a more complete pattern of its biological and toxicological activities. We performed an extensive analysis of the toxicological profile of aruginosin 828A on the following basis:

1. The *Planktothrix* strain used in our study did not produce MCs (in contrast to other strains of the same species). The strains devoid of MCs but producing aeruginosins are believed to compensate for the lack of MC toxicity. To determine whether aeruginosin 828A really compensates for MCs—a hypothesis formulated in [11]—it was necessary to determine the whole spectrum of effects as found for MCs [7].
2. To determine the similarity or dissimilarity of aeruginosin 828A to CP1020, we evaluated the same genes that were shown to be affected by CP 1020 in zebrafish, as shown in our previous study [20].
3. To screen for additional unknown toxicological effects, we determined important toxicological pathways in order to obtain a more complete pattern of biological action of aeruginosin 828A. Only a thorough investigation covering diverse potential endpoints allows the description of a more complete toxicological profile of the compound.

To this end, we compared the activities in human hepatoma cells Huh7, zebrafish eleuthero-embryos and in liver organ cultures. We demonstrated the anti-inflammatory activity of AG 828A in stimulated Huh7 cells, and further affirmed this finding with another cyanobacterial protease inhibitor, CP 1020. Furthermore, we found a different mode of action of AG 828A, as compared to MC-LR, both in zebrafish eleuthero-embryos and in liver organ cultures. The cyclic peptides of the MC family are non-ribosomal peptides and strong inhibitors of eukaryotic phosphatases [22], and they have additional molecular effects. In contrast to MC-LR, ER stress induction was not found following exposure to AG 828A in zebrafish liver organ cultures. Additionally investigated estrogenic effects resulting in vitellogenin induction, which have been found for cyanobacterial crude extracts [4], were not found for aeruginosin in the present study. No vitellogenin induction was found in zebrafish eleuthero-embryos and liver organ cultures after exposure to this aeruginosin. The search for additional toxicologically relevant activities of AG 828A did not reveal any other activity on the transcriptional level, although a series of diverse pathways were evaluated. This leads to the conclusion that AG 828A has a very restricted biological activity, namely anti-inflammatory action. General toxicologically relevant pathways and activities were not triggered by this cyanobacterial compound in Huh7 cells and zebrafish. 

The effect concentrations of aeruginosin 828A are pharmacologically rather than environmentally relevant. However, the uptake of aeruginosin 828A probably needs specific uptake transporters that may be expressed in Huh7 cells only to a low extent, thus potentially resulting in low concentrations of aeruginosin intracellularly. Further investigations using other cell lines, as well as uptake experiments would help so solve this issue. Moreover, it is not known whether aeruginosin 828A is accumulated in the liver of exposed fish and humans. If so, the effect concentrations may be of physiological relevance.

### 3.1. Anti-Inflammatory Activity in Huh7 Cells

TNFα contained in the cell culture medium of our experiments bound to the receptors on the cell surface and induced inflammation processes, including transcriptional up-regulation of target genes, such as *IL8* and *TNFα*. We demonstrated that AG 828A induced anti-inflammatory activity. Our data confirm the anti-inflammatory activity found in another aeruginosin variant, AG 865 [15]. By exposure of human lung microvascular endothelial cells to AG 865, down-regulation of *IL8* was observed [15], and this variant was found to be a noteworthy immunomodulatory agent, and the first aeruginosin with this characteristic. Similar to AG 865, AG 828A is not cytotoxic and no general toxicological pathways seem to be influenced by this compound. This makes AG 828A an additional interesting aeruginosin in regard to anti-inflammatory activity. The anti-inflammatory action found in our study for AG 828A could be based on preventing TNFα from binding to the receptors, or by intracellular actions within the signaling cascade. The anti-inflammatory action and its basis need further investigation. While AG 828A had anti-inflammatory activity on the transcriptional level, MC-LR was implicated in inflammatory activity [6]. This points to a significant difference in the mode of action of the phosphatase inhibiting microcystin, and the protease inhibiting AG 828A.

### 3.2. Up-Regulation of CYP1A in Huh7 Cells

The cytochrome P450 family of enzymes is responsible for biotransformation of a vast number of compounds. The induction of *CYP1A* in the liver is regulated by ligand binding to the AHR. Exposure of Huh7 cells to AG 828A in HBSS buffer resulted in a dose-dependent up-regulation of *CYP1A* transcription. This up-regulation was significant at 50 and 100 µmol·L^−1^. The structural element of all aeruginosins is choi, an indole-containing structural element [13]. Indol-containing compounds are known to weakly induce *CYP1A* by binding of the AHR. However, the transcriptional induction effect found following exposure in HBSS buffer was lacking in experiments, where we used culture media without FBS. HBSS is not optimally buffered for exposure at our culture conditions (5% CO_2_), resulting in a slight pH decrease over time. The exposure media were chosen with respect to the zebrafish liver organ culture experimental setup (but not performed at 5% CO_2_). The slight change in pH may have changed the structure of AG 828A. In acidic conditions, protonation can occur, which does not happen in neutral pH [11]. A positively charged compound could undergo a different uptake by specific transporters, and as a consequence, influence the effects of AG 828A.

However, in our EROD assay no induction occurred. We analyzed EROD activity after exposure to AG 828A under various conditions. According to the protocol of [24] we used medium with FBS. However, as the concentration of AG 828A could have been lower due to potential binding to FBS, we also analyzed EROD activity by use of serum free cell culture media. However, neither of the different experimental settings, using different exposure media, resulted in induction of EROD activity. Therefore *CYP1A* induction on the transcription level could not be demonstrated on the protein level. Nevertheless, the results are interesting, and further investigations are needed to explain the *CYP1A* transcriptional induction in Huh7 cells by AG 828A in HBSS buffer. 

### 3.3. Effects in Zebrafish Eleuthero-Embryos and Liver Organ Cultures

AG 828A and CP 1020 are both protease inhibitors and showed similarly acute toxicity to *Thamnocephalus platyurus* [11,16]. Furthermore, we showed in our study that AG 828A and CP 1020 seem to have similar anti-inflammatory activity. In zebrafish embryos, however, effects differ. Transcriptional changes induced by CP 1020 [20] were not found after exposure to similar concentrations of AG 828A in our present study. 

No molecular effects were found by the targeted gene approach covering a considerable series of different effect pathways in zebrafish eleuthero-embryos or zebrafish liver organ culture after exposure to AG 828A. The ER stress induction was analyzed to compare the effects of AG 828A to microcystin, as AG 828A was suggested as a replacement for the loss of microcystin [11]. Microcystin-LR was shown to induce ER stress in zebrafish liver organ cultures [7]. ER stress activates the unfolded protein response and occurs after accumulation of unfolded proteins resulting in the expressional up-regulation of several marker genes, including *bip* and *chop*. Furthermore, as a consequence, splicing of *xbp-1*-mRNA is induced [6]. However, no alteration in ER stress marker gene transcripts by AG 828A was found in zebrafish embryos and zebrafish liver organ cultures.

Estrogenic activity was found in cyanobacterial crude extracts, but the substances responsible could not be identified [4]. For this reason, we analyzed AG 828A for its estrogenic activity by evaluation of transcriptional up-regulation of *vtg*, a yolk precursor. However, no transcriptional induction of vitellogenin was found and therefore the compound responsible for this estrogenic activity is not related to AG 828A. Further cyanobacterial toxins should be analyzed for estrogenicity, to identify the compound responsible for this activity.

## 4. Conclusions

This study showed that AG 828A and CP 1020 have similar anti-inflammatory effects in stimulated Huh7 cells. However, effects of these cyanobacterial toxins differ in zebrafish eleuthero-embryos and in the liver. Microcystin-LR also differed from AG 828A with respect to ER stress in zebrafish liver, where AG 828A showed no activity. Furthermore, no estrogenic activity or any other significantly affected pathway was identified as a molecular effect of AG 828A. Further investigations are needed to clarify the anti-inflammatory action of AG 828A, including its particular mode of action. Similar investigations should be focused on CP 1020. Furthermore, future research directions may also be devoted to the role of active uptake transporters to mediate uptake of AG 828A and CP 1020.

## 5. Materials and Methods

### 5.1. Culture of Cyanobacteria and Isolation of AG 828A and CP 1020

For the isolation of AG 828A, *Planktothrix rubescens* strain 91/1 was cultured as previously described [10]. For this purpose, 300 mL Erlenmeyer flasks containing 120 mL mineral medium [24] was used at a constant temperature of 20 °C, as well as constant light conditions at an irradiation of 6 µmol/(ms)^2^ from fluorescent tubes (Osram 930; Lumilux Delux; Warm White 3000 K, Munich, Germany). The isolation of AG 828A was also performed in a similar way to the procedure of [11]. *Planktothrix* cells were harvested by centrifugation at 20,000 relative centrifugal force (RCF) for 20 min (Beckman Coulter Avanti 3-25I Ultracentrifuge, Beckman Coulter GmbH, Krefeld, Germany), and after a freeze/thaw cycle, AG 828 was extracted with 50% methanol (Sigma Aldrich, Buchs, Switzerland) (10 mL per gram biomass) in the dark. After centrifugation at 20,000 RCF for 20 min, the supernatant was filtered (Machery-Nagel, 713 ¼, Machery-Nagel GmbH & Co. KG, Düren, Germany), and solvent was evaporated to dryness in a GeneVac EZ-2 Plus (Stepbios, Muttenz, Switzerland). The dried extract was resolved in 60% methanol and AG 828 purified according to [11] with some modifications. 

The reverse phase high-performance liquid chromatography (HPLC) instrument consisted of a degasser, quaternary pump, autosampler, column oven and diode array detector connected to an analytical-scale fraction collector (Agilent Series 1100, Agilent Technologies, Waldbronn, Germany). The separation of the cultured extract was performed with a reversed phase column YMC Hydrosphere C_18_ (4.6 mm × 250 mm, 5 micro) (Stagroma, Reinach, Switzerland), and the following mobile phases: Water (A) and acetonitrile (B) (Sigma Aldrich, Buchs, Switzerland) both containing 0.05% trifluoroacetic acid (TFA) (Sigma Aldrich, Buchs, Switzerland). A linear gradient of 25% to 100% B in 10 min was applied at a flow rate of 1 mL per minute. The column compartment was maintained at 30 °C and the detection wavelength was 250 nm (16 nm band-width) with a reference wavelength of 360 nm (100 nm band-width) by 2.5 Hz sampling rate. UV spectra were acquired in 190–500 nm range in 4 nm steps. 

The fraction that eluted between 7.5 and 8.8 min was collected in 20 mL vessels in many analytical runs and concentrated with the GeneVac system (Stepbios, Muttenz, Switzerland). The concentrated eluate was further separated on an Ascentis Express C_18_ (4.6 mm × 150 mm, 2.7 µm) (Sigma Aldrich, Buchs, Switzerland). The mobile phase was water for channel A and methanol (Sigma Aldrich, Buchs, Switzerland) for channel B, both without TFA (Sigma Aldrich, Buchs, Switzerland). Starting at 45% B for 0.5 min and with a subsequent linear gradient to 80% B in 7.5 min, aeruginosin 828A eluted at 5.8 min and was analysed using high-resolution mass spectrometry (Figures S12–S14, Table S1) (see supplementary for method details).

For the isolation and purification of CP 1020, *Microcystis aeruginosa* UV-006 was cultured as previously described [16], and cells were harvested by centrifugation at 10,000 *g* using a 6K15 centrifuge (Sigma Aldrich, Buchs, Switzerland). Subsequently, peptides were extracted with 60% acetonitrile (10 mL per gram cyanobacterial biomass), and three times one minute of sonication. Extracts were centrifuged at 10,000 *g* in a 6K15 centrifuge (Sigma Aldrich, Buchs, Switzerland), and vacuum evaporation was applied afterwards using a rotatory evaporator (Büchi, Flawil, Switzerland) to remove the solvents from the supernatant. After resolving in 60% acetonitrile (1 mL per gram cyanobacterial biomass), CP 1020 was purified by preparative HPLC using a Dionex P-680 HPLC System (Dionex, Sunnyvale, CA, USA) with a Phenomenex Gemini-NX C18 5 μ (21.2 mm × 75 mm) column using a linear gradient of 5% to 100% acetonitrile in 0.1% formic acid/water over 40 min at a flow rate of 5 mL·min^−1^. CP 1020 eluted after 14.4 min. Formic acid was removed from the eluate using a C18 Cartridge (10 g; Mega Bond Elute, Agilent Technologies, Waldbronn, Germany). The cartridge was conditioned with 60% acetonitrile (Sigma Aldrich, Buchs, Switzerland), and after adding the eluate, it was flushed several times with water before CP 1020 was eluted with 100% acetonitrile. After removal of all volatiles (rotarory evaporator, Büchi, Flawil, Switzerland) and lyophilisation pure CP 1020 was afforded and measured using high-resolution mass spectrometry (Figures S15–S17) (see supplementary for method details).

### 5.2. Zebrafish

Husbandry of adult zebrafish was performed as previously described [25]. For egg production, a spawning tray was used consisting of a stainless steel tray, a grid covered with stones as well as a plastic plant for spawning stimulation. These trays were transferred in the evening into 10 L tanks, containing a small group of fish. The next morning after the onset of light, spawned eggs fell through the grid of the spawning tray, and were taken out 1–2 h later and washed with reconstituted fish water. Reconstituted fish water was prepared as followed: Deionized water with ions added (CaCl_2_ × 2H_2_O 147.0 mg·L^−1^, KCl 2.9 mg·L^−1^, MgSO_4_ × 7H_2_O 61.6 mg·L^−1^, NaHCO_3_ 32.4 mg·L^−1^) (Sigma Aldrich, Buchs, Switzerland). Egg quality was checked under the stereo-microscope (Carl Zeiss AG, Feldbach, Switzerland).

### 5.3. Culture of Human Hepatoma Cell Line Huh7

Huh7 cells were kindly provided by M. Heim, University Hospital Basel, Switzerland. Cells were cultured in DMEM/Glutamax (LuBioScience, Lucerne, Switzerland) with 10% fetal bovine serum (FBS) (Sigma Aldrich, Buchs, Switzerland) and 1% Penicillin-Streptomycin (LuBioScience, Lucerne, Switzerland) was added to the media. Cells were grown in an QWJ 300T/BB incubator (Brouwer, Luzern, Switzerland) at 37 °C and 5% CO_2_. 

### 5.4. Anti-Inflammatory Activity Assessment of AG 828A in Huh7 Cells

Huh7 cells were seeded on a 48 well plate (Huberlab, Reinach, Switzerland) and grown to 70% confluency. Cells were pretreated for 30 min with AG 828A and CP 1020 and then 1 ng·L^−1^ TNFα (Enzo Lifescience, Lausen, Switzerland) was added. The experimental design was similar to [15]. A 30 min pre-treatment with the potential anti-inflammatory peptides AG 828A and CP 1020, and a co-treatment with these peptides together with TNFα as inflammatory stimulus for 24 h. DMSO (Sigma Aldrich, Buchs, Switzerland) was used to dissolve the peptides and each concentration group contained 1% DMSO. Exposure groups included a negative control without TNFα, a control with TNFα only, a low and high concentration (50 µmol·L^−1^ and 100 µmol·L^−1^) of AG 828 A, respectively, and a group containing 100 µmol·L^−1^ CP 1020. Exposure was performed in Huh7 cell culture medium without FBS. After 24 h RNA was extracted, cDNA synthesized and RT-qPCR was performed (see Section 5.9).

### 5.5. Transcriptional Effects of AG 828A in Huh7 Cells

Two different settings were used. In both settings, cells were seeded in 48 well plates (Huberlab, Reinach, Switzerland) (100,000 cells per well) in culture medium. After 24 h, cells were treated with AG 828 A. In a first setting, exposure was performed in HBSS buffer (LuBioScience, Lucerne, Switzerland) with concentrations of 0, 5, 20, 50, and 100 µmol·L^−1^ AG 828 A, with 1% DMSO (Sigma Aldrich, Buchs, Switzerland) in each concentration group. In a second setting, concentrations of 0, 25, 50, and 100 µmol·L^−1^ were prepared in culture medium without FBS. After 24 h of exposure at 37 °C and 5% CO_2_, RNA was extracted, cDNA was synthesized and RT-qPCR was performed (see Section 5.9). Primer sequences are listed in Tables S2 and S3.

### 5.6. EROD Assay in Huh7 Cells

EROD assay was performed to measure cytochrome P450 1A (CYP1A) activity. This assay is based on the conversion of 7-ethoxyresorufin to the fluorescent resorufin by ethoxyresorufin-O-deethylase. The amount of product and therefore activity of CYP1A is determined by fluorescence measurements. Cells were seeded on 96 well plates in culture medium (50,000 cells on a black plate with clear bottom, Becton Dickinson, Allschwil, Switzerland). A 24 h exposure was performed in a first setting at 0, 20, 50, and 100 µmol·L^−1^ AG 828A in medium with FBS. As a positive control, the known CYP1A inducer benzo[*a*]pyrene (BaP) (Sigma Aldrich, Buchs, Switzerland) was used in concentrations of 0.1 and 1 µmol·L^−1^, as well as the CYP1A and ER stress inducer tunicamycin (Enzo Lifescience, Lausen, Switzerland) at 12 µmol·L^−1^ [26]. In a second setting, exposure was performed using 50 µmol·L^−1^ AG 828A and 1 µmol·L^−1^ BaP in HBSS (LuBioScience, Lucerne, Switzerland), or 1 µmol·L^−1^ BaP (Sigma Aldrich, Buchs, Switzerland) in medium with FBS (Sigma Aldrich, Buchs, Switzerland). Solvent controls of 1% DMSO (Sigma Aldrich, Buchs, Switzerland) were also included.

After 24 h, the exposure media were replaced by HBSS (LuBioScience, Lucerne, Switzerland) containing 1 µmol·L^−1^ of the CYP1A substrate 7-ethoxyresorufin (Fluka, Buchs, Switzerland) and plates were incubated for 20 min at culture conditions. A Synergy 2 plate reader (Biotek instruments, Lucerne, Switzerland) was used to measure the fluorescence of the product resorufin using Gen5 software (version 1.08, Biotek instruments, Lucerne, Switzerland, 2005). The exciatation wavelength was 544 nm, the detected emission wavelength 590 nm. 

### 5.7. Exposure of Zebrafish Eleuthero-Embryos for Gene Expression Analysis

At 72 h post fertilization (hpf), hatched zebrafish embryos were transferred to 48 well plates (one eleuthero-embryo per well) (Huberlab, Reinach, Switzerland) containing reconstituted fish water. The water was subsequently replaced by 450 µL of exposure medium, which was reconstituted fish water containing AG 828A at concentrations of 0, 100, and 1000 µg·L^−1^. DMSO (Sigma Aldrich, Buchs, Switzerland) was used as solvent with a final concentration of 0.01% in every concentration group. Each group consisted of 4 replicates with 15 eleuthero-embryos each. Eleuthero-embryos were statically exposed until 168 hpf and fixed in RNA Later (Qiagen, Basel, Switzerland) afterwards. RNA was extracted, cDNA synthesized, and RT-qPCR was performed (see Section 5.9). The same primers were used as in [7,20].

### 5.8. Zebrafish Liver Organ Culture and Exposure to AG 828A for Gene Expression Analysis

The isolation of liver of seven adult female zebrafish, followed by a subsequent 5 h exposure to compounds was performed according to [7]. Female zebrafish were euthanized in Koi med sleep (Schönbach Apotheke, Asslar, Germany) and liver was dissected, divided and distributed to four concentration groups. Liver pieces were exposed to 0, 0.1 and 1 µmol·L^−1^ AG 828A, and 1 µmol·L^−1^ tunicamycin (Enzo Lifescience, Lausen, Switzerland) as a positive control. Exposure groups were prepared in HBSS buffer (LuBioScience, Lucerne, Switzerland), and a solvent concentration of 0.5% DMSO (Sigma Aldrich, Buchs, Switzerland). Tunicyamycin (Enzo Lifescience, Lausen, Switzerland) is a known ER stress inducer and was furthermore found to induce CYP1A [27]. Afterwards, the liver was fixed in RNA Later (Qiagen, Basel, Switzerland), RNA was extracted, cDNA synthesized, and qRT-PCR performed (see Section 5.9). The same primers were used as in [7]. For data processing of liver organ cultures, each liver was analyzed separately and transcriptional changes after exposure to AG 828A were normalized to the respective control piece. 

### 5.9. Total RNA Extraction, cDNA Synthesis and RT-qPCR

Total RNA of Huh7 cells was isolated using TRIZOL (Fischer Scientific, Reinach, Switzerland) according to the manufacturer’s instructions. The dried RNA pellet was resuspended in 20 µL of RNAse free water (Qiagen, Basel, Switzerland). Total RNA from zebrafish tissue (liver or zebrafish eleuthero-embryos) was extracted using RNeasy Mini Kit (Qiagen, Basel, Switzerland) according to the manufacturer’s instructions. RNA concentrations and purity were measured spectrophotometrically (Nanodrop ND-1000, Witec AG, Lucerne, Switzerland using ND1000 software, version 3.8.1), and single strand cDNA synthesis and RT-qPCR were performed as previously described [20].

### 5.10. Statistics

All data were processed with GraphPad Prism software (Version 5, GraphPad Software, Inc., La Jolla, CA, USA, 2007) and statistics were performed using One-way ANOVA (with Dunett post test).

## Figures and Tables

**Table 1:** List of genes analyzed in Huh7 cells, zebrafish eleuthero-embryos and zebrafish liver organ cultures exposed to aeruginosin 828A.

Pathway	Gene symbol	Gene name
**Huh7 cells**		
Aryl hydrocarbon receptor (AHR) regulated genes	*CYP1A*	*Cytochrome P450 1A*
	*AHR*	*Aryl hydrocarbon receptor*
	*GSTa1*	*Glutathione S-Transferase a1*
	*UGT1A6*	*Uridine diphospho-glucuronosyltransferase 1A6*
	*ODC*	*Ornithin-decarboxylase*
	*NQO*	*Chinon oxidoreductase*
Endoplasmic reticulum (ER) stress	*BIP*	*Binding immunoglobulin protein*
	*CHOP*	*CCAAT/enhancer binding protein (C/EBP) homologous protein*
	*XBP-1s*	*Spliced X-box binding protein 1*
	*GADD34*	*Growth arrest and DNA damage-inducible protein 34*
	*DR5*	*Death receptor 5*
Mitogen-activated protein kinase (MAPK) signaling	*CJUN*	*C-Jun N-terminal kinase*
	*CFOS*	*CFOS*
Apoptosis	*CASP8*	Caspase 8
Inflammation	*IL8*	*Interleukin 8*
	*TNFα*	*Tumor necrosis factor α*
Urokinase activation system	*uPA*	*Urokinase, plasminogen activator*
	*PAI*	*Plasminogen activator inhibitor type 1*
**Zebrafish embryos**		
ER stress	*bip*	*Binding immunoglobulin protein*
	*chop*	*C/EBP homologous protein*
	*xbp-1s*	*Spliced X-box binding protein 1*
Oxidative stress	*cat*	*Catalase*
MAPK signaling	*cjun*	*C-Jun N-terminal kinase*
Apoptosis	*p53*	*Tumor suppressor protein p53*
DNA damage	*cry5*	*Cryptochrome 5*
Inflammation	*tnfα*	*Tumor necrosis factor α*
Estrogen signaling	*vtg*	*Vitellogenin*
	*esr*	*Estrogen receptor*
	*cyp19b*	*Cytochrome P450 19b*
Hypothalamic-Pituitary-	*tshβ*	*Thyroid-stimulating hormone*
Thyroid Axis		
Ahr regulated	*cyp1a*	*Cytochrome P450 1A*
Circadian rhythm	*nr1d1*	*Nuclear receptor subfamily 1, group D, member 1*
	*per1*	*Period 1*
DNA damage response	*cry5*	*Cryptochrome 5*
Adenosine triphosphate-(ATP) binding cassette transporter	*abcg2*	*ATP-binding cassette sub-family G member 2*
Prostaglandin synthesis	*ptgds*	*Prostaglandin D2 synthase*
**Zebrafish liver**		
Ahr regulated	*ahr1*	*Aryl hydrocarbon receptor 1*
	*ahr2*	*Aryl hydrocarbon receptor 2*
	*cyp1a*	*Cytochrome P450 1A*
ER stress	*bip*	*Binding immunoglobulin protein*
	*chop*	*C/EBP homologous protein*
MAPK signaling	*cjun*	*C-Jun N-terminal kinase*
	*dusp5*	*Dual specificity phosphatase 5*
Oxidative stress	*cat*	*Catalase*
	*sod*	*Superoxid dismutase*
Apoptosis	*casp3*	*Caspase 3*
	*bax*	*Bcl-2-like protein*

## Supplementary Materials

The Supplementary Materials are available online at www.mdpi.com/2072-6651/8/7/219/s1.
