# Supplementary material for: Anti-Inflammatory Activity of Cyanobacterial Serine Protease Inhibitors Aeruginosin 828A and Cyanopeptolin 1020 in Human Hepatoma Cell Line Huh7 and Effects in Zebrafish (Danio rerio)
Source: Toxins (Basel). 2016 Jul 14;8(7):219. doi: 10.3390/toxins8070219 (PMC4963851; doi:10.3390/toxins8070219)
Supplement: Supplementary file 1 [file toxins-08-00219-s001.pdf]

Supplementary Materials: Anti-Inflammatory Activity of Cyanobacterial Serine Protease Inhibitors Aeruginosin 828A and Cyanopeptolin 1020 in Human Hepatoma Cell Line Huh7 and Effects in Zebrafish (*Danio rerio*)

Susanne Faltermann, Simon Hutter, Verena Christen, Timm Hettich and Karl Fent

Transcriptional Analysis in Huh7 Cells after Exposure to Different Concentrations of AG 828A in HBSS Buffer (Figures S1–S3)

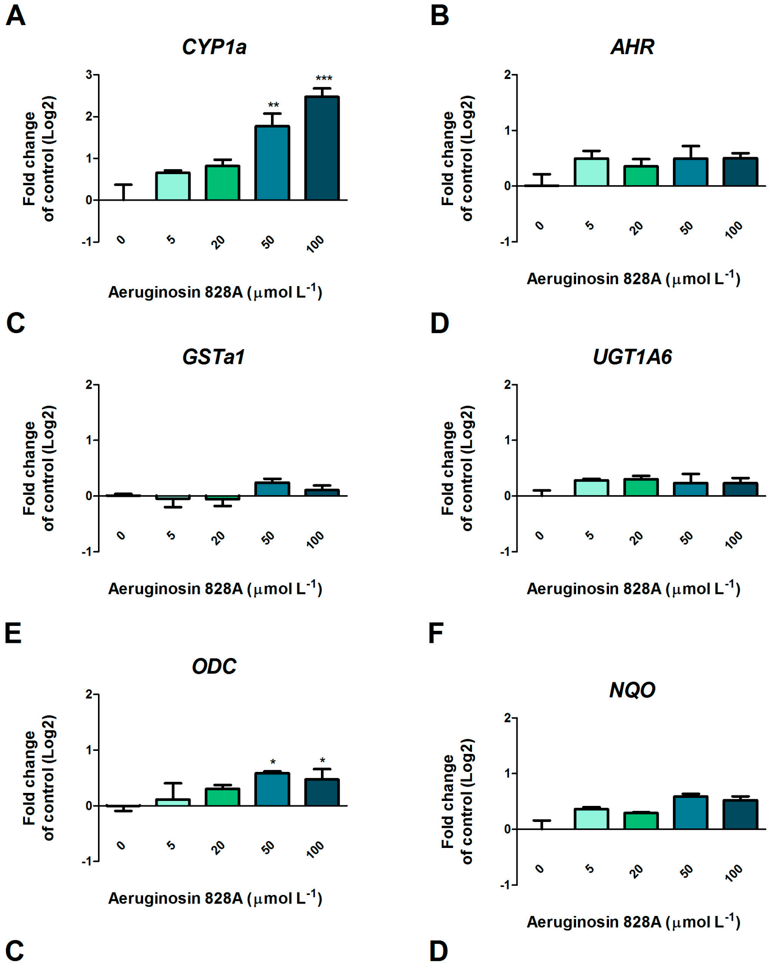

Figure S1. Transcription expression of aryl hydrocarbon receptor (AHR) regulated genes in Huh7 cells exposed to AG 828A in HBSS buffer compared to cells. (**A**) *Cytochrome P450 1A* (*CYP1A*); (**B**) *Aryl hydrocarbon receptor* (*AHR*); (**C**) *Glutathione S-Transferase a1* (*GSTa1*); (**D**) *Uridine diphospho-glucuronosyltransferase 1A6 (UGT1A6)*; (**E**) *Ornithin-decarboxylase* (*ODC*); (**F**) *nicotinamid adenindinucleotide phosphate* (*NADPH*) *chinon oxidoreductase* (*NQO*). Significant changes compared to control are indicated by asterisks (* $p < 0.05$; ** $p < 0.001$; *** $p < 0.0001$).

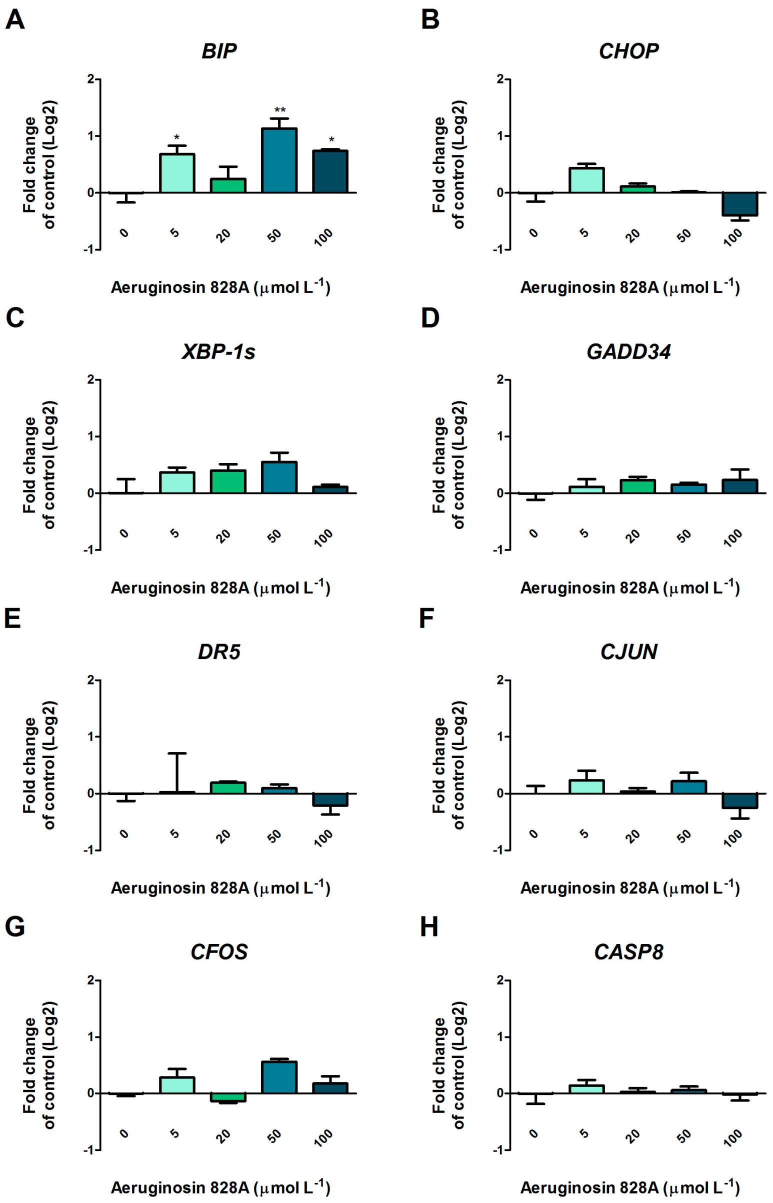

Figure S2. Transcriptional expression of genes that are known to be influenced by microcystin exposure in Huh7 cells exposed to AG 828A in Hank's Balanced Salt Solution (HBSS) buffer compared to control cells. (**A**) *Binding immunoglobulin protein* (*BIP*); (**B**) *CCAAT/enhancer binding protein (C/EBP) homologous protein* (*CHOP*); (**C**) *Spliced X-box binding protein 1* (*XBP1-*); (**D**) *growth arrest and DNA damage-inducible protein 34* (*GADD34*); (**E**) *Death receptor 5* (*DR5*); (**F**) *jun proto-oncogene* (*CJUN*); (**G**) *fos proto-oncogene* (*CFOS*); (**H**) *caspase 8* (*CASP8*). Significant changes compared to control are indicated by asterisks (* $p < 0.05$; ** $p < 0.001$).

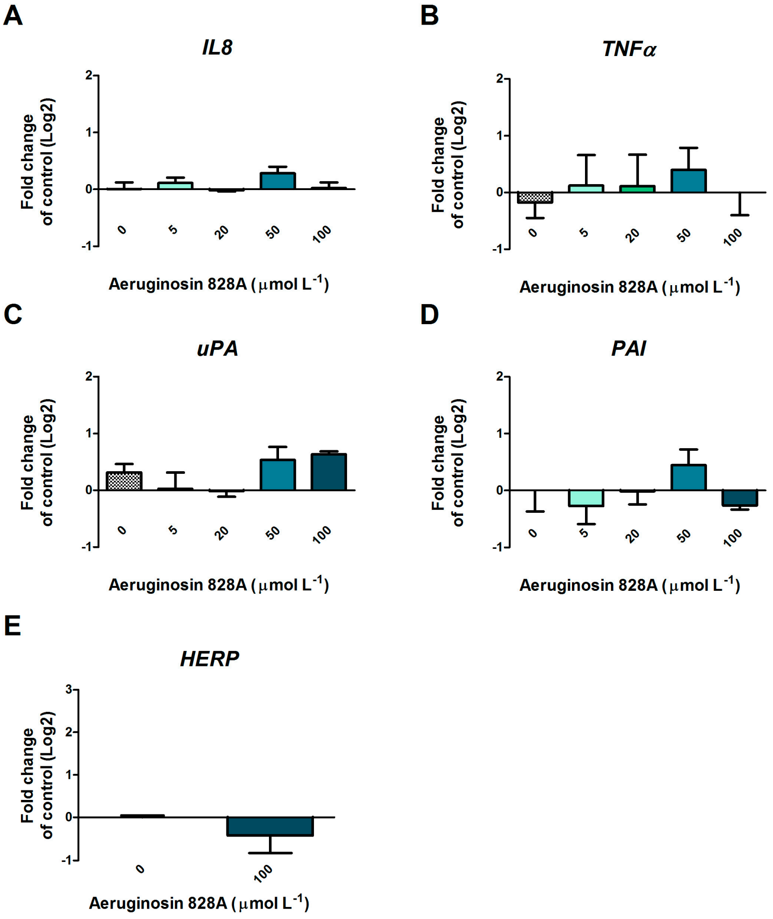

Figure S3. Transcriptional expression of genes involved in inflammation (**A**,**B**); the urokinase activation system (**C**,**D**) and endoplasmic reticulum (ER) stress (**E**) in Huh7 cells exposed to AG 828A in HBSS buffer compared to control cells. (**A**) *Interleukin 8* (*IL8*); (**B**) *Tumor necrosis factor α* (*TNF α*); (**C**) *Urokinase plasminogen activator* (*uPA*); (**D**) *Plasminogen activator inhibitor type 1* (*PAI*); (**E**) *Homocysteine inducible ER protein with ubiquitin like domain 1* (*HERP*).

Transcriptional Analysis in Zebrafish Embryos after Exposure to Different Concentrations of AG 828A (Figures S4–S6)

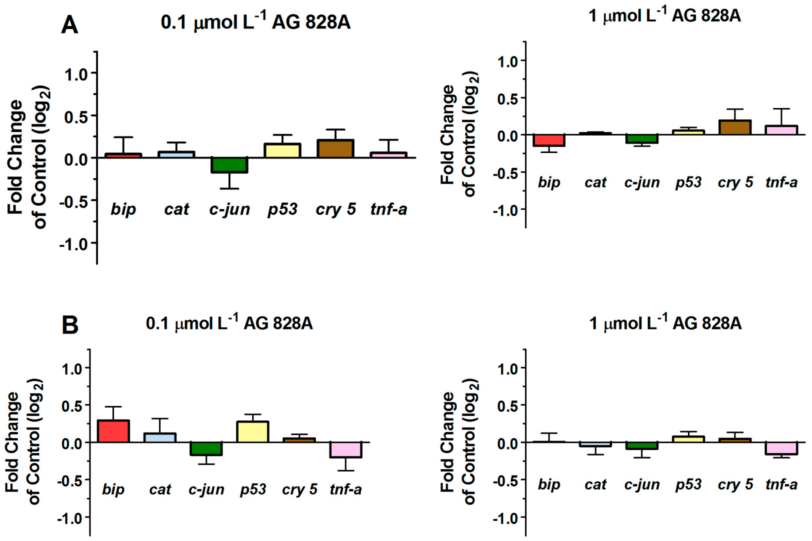

Figure S4. Transcription of genes involved in cellular stress responses (*bip*, *catalase* (*cat*), *c-jun*, *tumor suppressor protein 53* (*p53*), *cryptochrome 5* (*cry5*), *tnfα*) in zebrafish eleuthero-embryos exposed to AG 828A compared to control eleuthero-embryos. (**A**) 48 h exposure; (**B**) 96 h exposure.

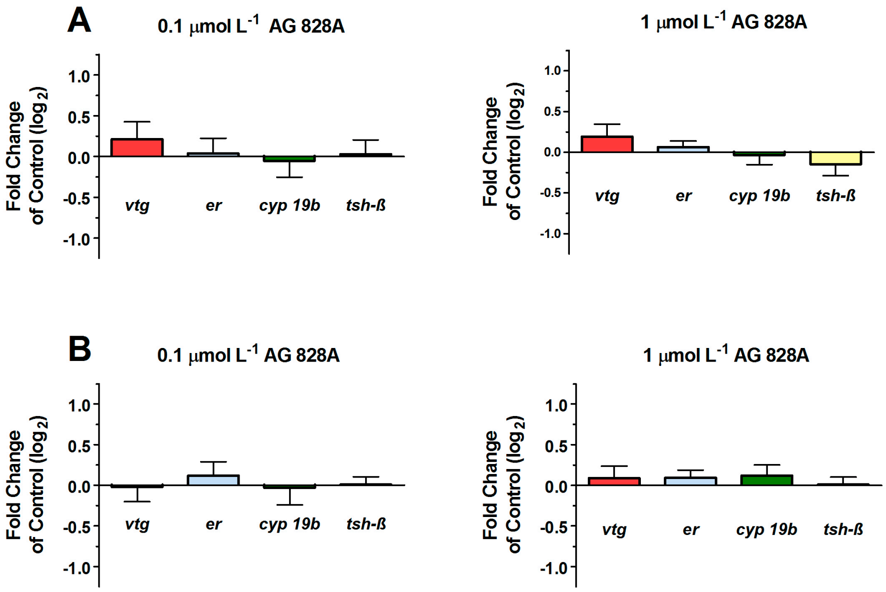

Figure S5. Transcription of genes involved in hormonal pathways (*vitellogenin* (*vtg*), *estrogen receptor* (*er*), *cytochrome P450 19b* (*cyp 19b*), *thyroid-stimulating hormone* (*tshβ*)) in zebrafish eleuthero-embryos exposed to AG 828A compared to control eleuthero-embryos. (**A**) 48 h exposure; (**B**) 96 h exposure.

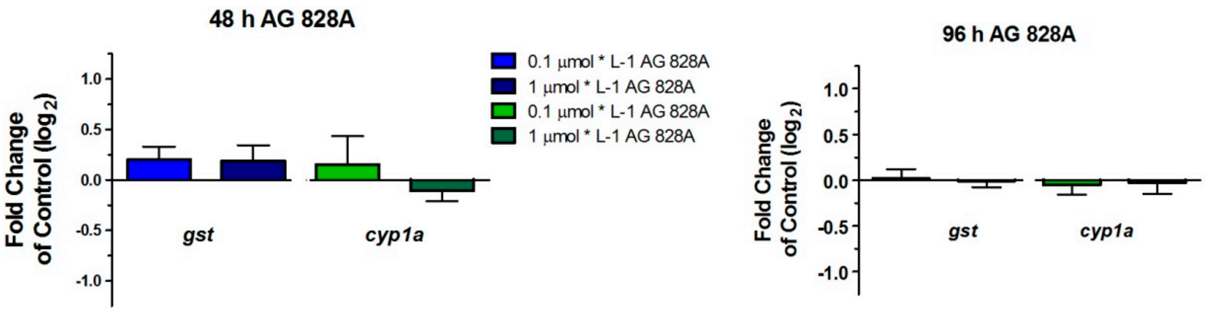

Figure S6. Transcription of genes involved in detoxification (*gst*, *cyp1a*) in zebrafish eleuthero-embryos exposed to AG 828A compared to control eleuthero-embryos for 48 h and 96 h exposure.

Transcriptional Analysis in Zebrafish Liver Organ Culture after Exposure to Different Concentrations of AG 828A and the ER Stress Inducer Tunicamycin (Figures S7–S11)

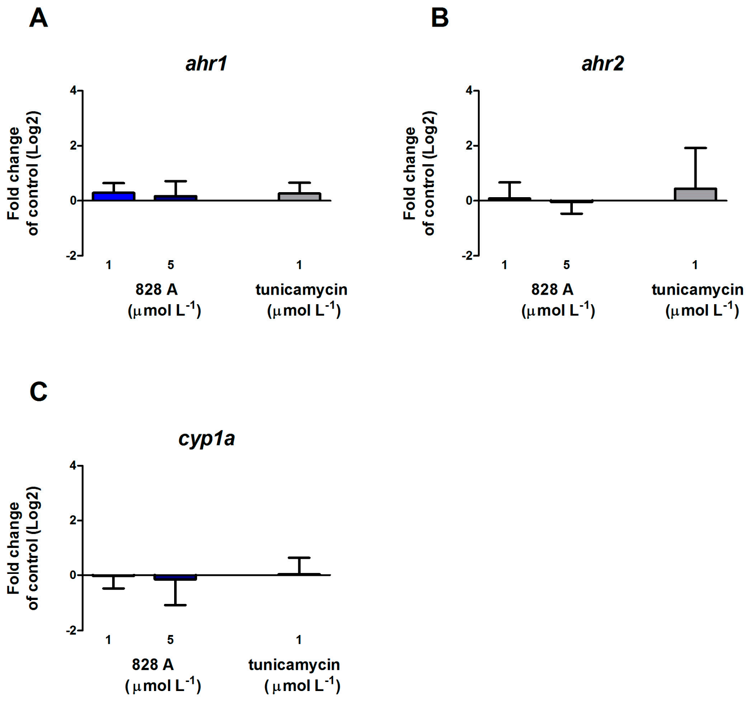

Figure S7. Transcriptional expression of AHR regulated genes in zebrafish liver organ cultures exposed to AG 828A for 5 h compared to control. (**A**) *Aryl hydrocarbon receptor 1*; (**B**) *Aryl hydrocarbon receptor 2*; (**C**) *Cytochrome P450 1A*.

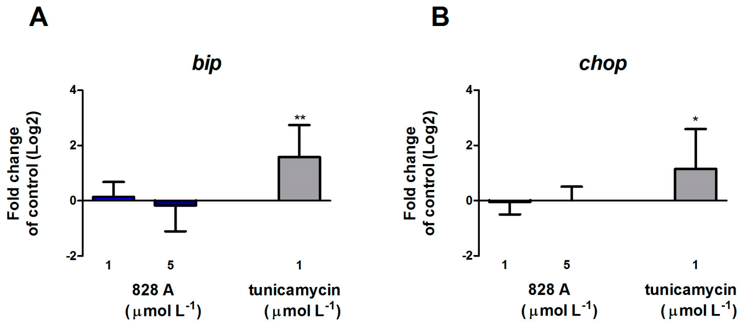

Figure S8. Transcriptional expression of genes involved in ER stress in zebrafish liver organ cultures exposed to AG 828A for 5 h compared to control. (**A**) *Binding immunoglobulin protein*; (**B**) *C/EBP homologous protein*. Significant changes compared to control are indicated by asterisks (* $p < 0.05$; ** $p < 0.001$).

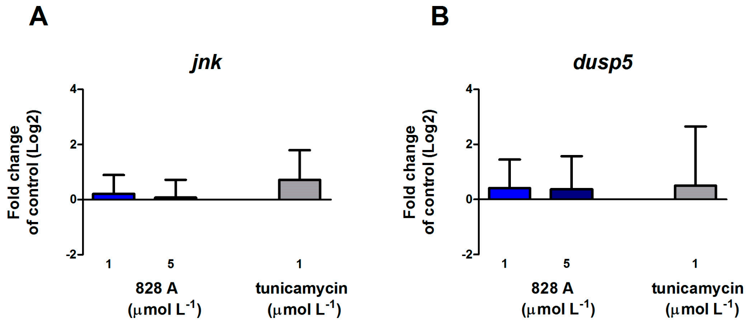

Figure S9. Transcriptional expression of genes involved in mitogen-activated protein kinase (MAPK) pathways in zebrafish liver organ cultures exposed to AG 828A for 5 h compared to control. (**A**) C-*Jun N-terminal kina (jnk)s*; (**B**) *Dual specificity phosphatase 5* (*dusp5*).

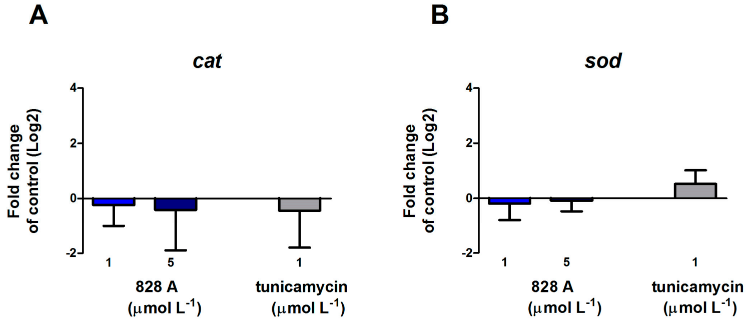

Figure S10. Transcriptional expression of genes involved in oxidative stress in zebrafish liver organ cultures exposed to AG 828A for 5 h compared to control. (**A**) *Catalase*; (**B**) *Superoxid dismutase* (*sod*).

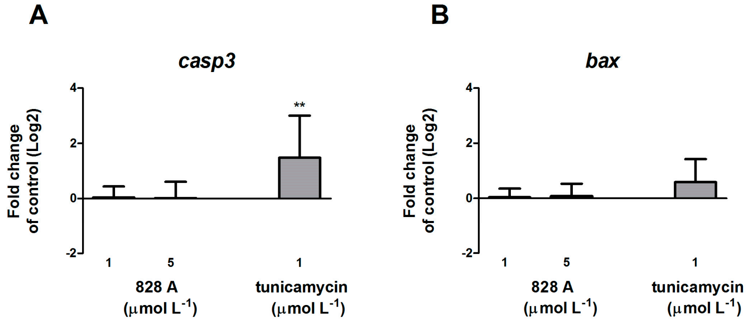

Figure S11. Transcriptional expression of genes involved in apoptosis in zebrafish liver organ culture exposed to AG 828A for 5 h compared to control. (**A**) *Caspase 3* (*casp3*); (**B**) *Bcl-2-like protein (bax*). Significant changes compared to control are indicated by asterisks (** $p < 0.001$).

Analysis of the Purified Aeruginosin 828A and CP 1020 is Described in the Following, and Results are Presented in Figures S12–S17 and Table S1

Chromatography separation was accomplished on a Scherzo SM C_{18} (2.1 × 100 mm, 3 micron, Imtakt USA, Portland, OR, USA) using an ultra high performance liquid chromatography stack arrangement consisting of a degasser, binary pump, auto sampler, thermostat and column oven (Agilent Series 1290, Agilent Technologies, Waldbronn, BW, Germany). A 1 µL aliquot of the purified sample was injected into the column and eluted with a linear gradient of 5%–95% solvent B over 4.5 min and kept at 95% B for 3 min and re-equilibration was performed in 3 min. Mobile phase A was water and mobile phase B was methanol plus each containing of 5 mM ammonium formate. The column was maintained at 40 °C and the column flow was set to 0.4 mL/min. Mass spectrometry spectra were assimilated on Q-TOF-MS system (Agilent Series 6540 Q-TOF, Agilent Technologies, Santa Clara, CA, USA) with a jet stream electrospray ion source (ESI). The ESI source was operated in positive mode with following parameter settings: nebulizer pressure 35 psig, nozzle voltage 0 V, sheath gas flow 11 L/min, sheath gas temperature 375 °C, drying gas flow 8 L/min, drying gas temperature 250 °C, capillary voltage 3000 V and fragmentor voltage 175 V respectively. Accurate mass spectra were acquired over an *m*/*z* of 100–1500 range by 8127 transitions per spectrum (1Hz). The acquired spectra were automatically recalibrated on-line by reference ions with exact masses 121.0509 and 922.0098 *m*/*z*. The system was running under the software MassHunter Acquisition and Qualitative Analysis version B.06.00 (Agilent Technologies, Santa Clara, CA, USA, 2012).

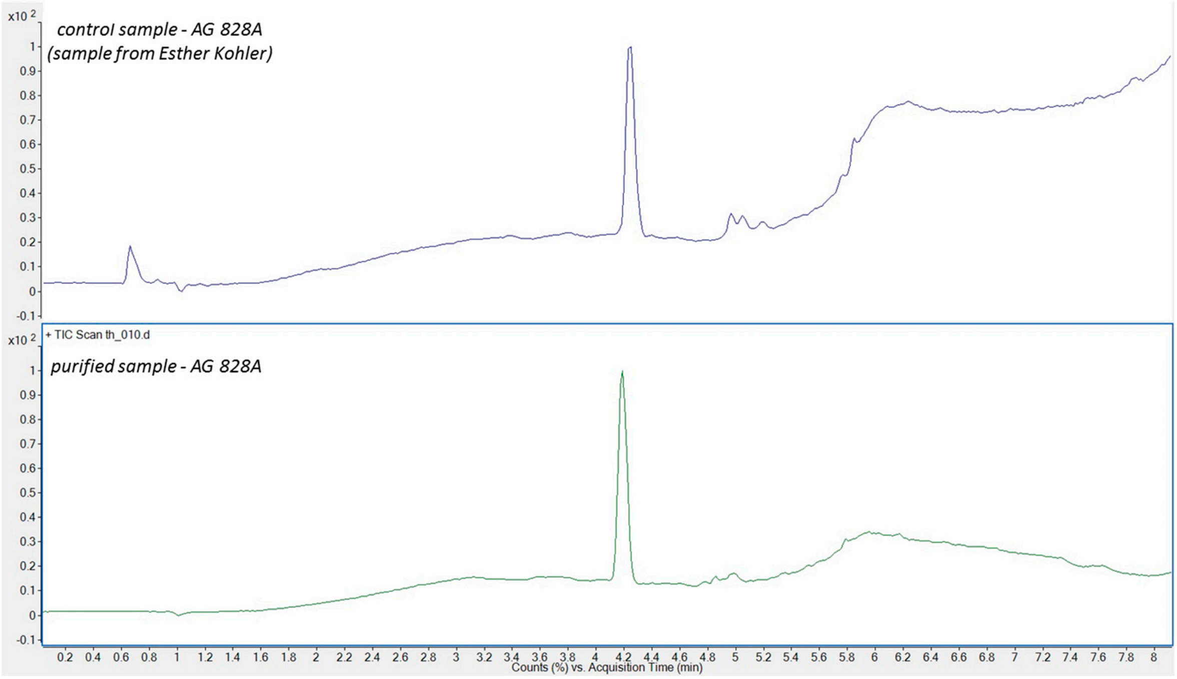

Figure S12. Total Ion Chromatogram of control sample AG 828A (provided from [14] as standard) and purified AG 828A of *Planktothrix rubescens* extract after the second fractionation step.

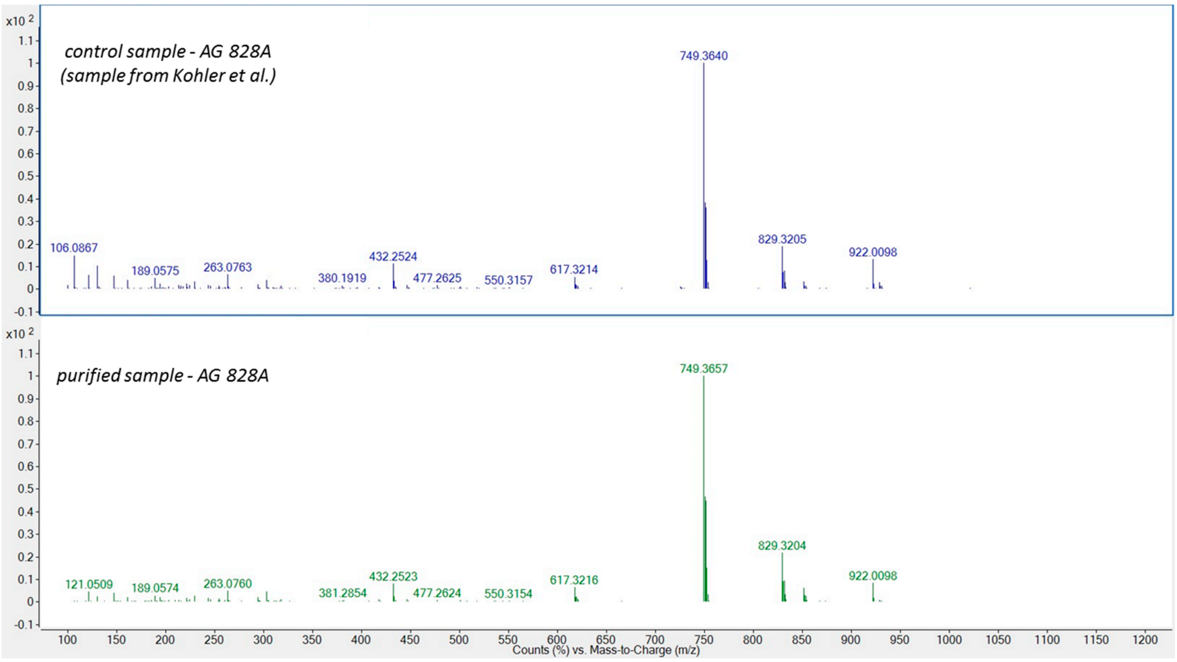

Figure S13. MS spectrum of AG 828A of the standard control sample (blue) and purified sample (green). Mass *m*/*z* 829 is the [M + H] signal and the base peak *m*/*z* 749 signify $[M + H - SO_3]^+$ of the targeted compound Ag 828A.

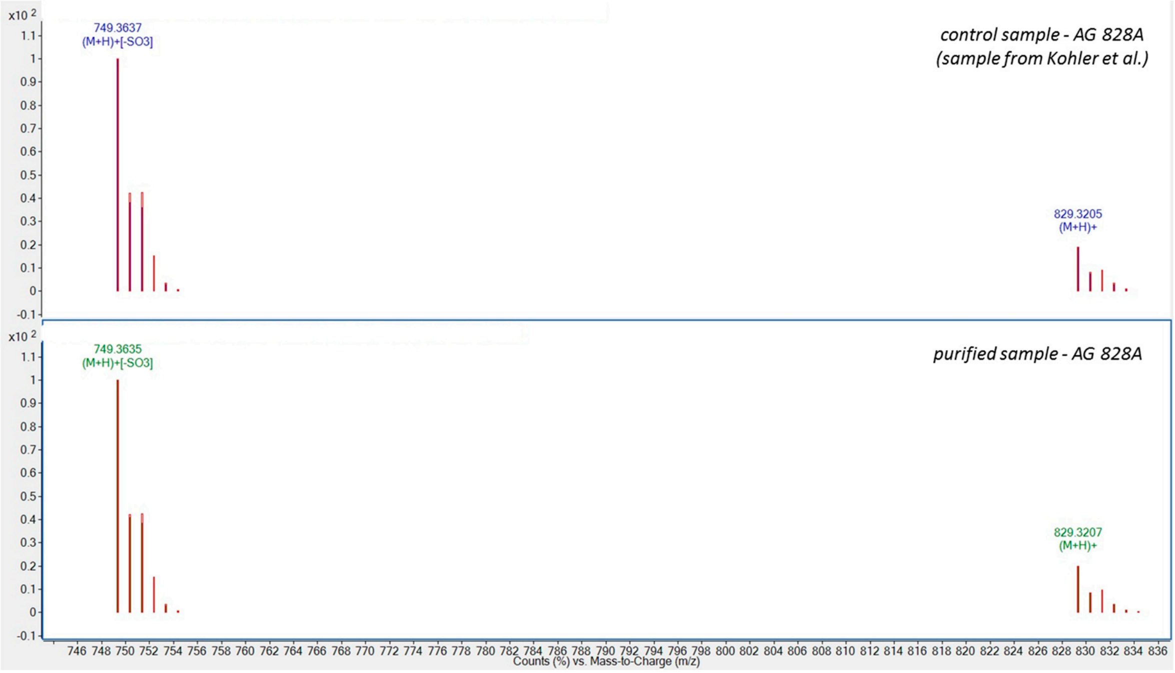

Figure S14. MS spectrum with charge carrier, neutral loss annotation and predicted isotope distribution (red bars) of AG 828A of the standard control sample and sample.

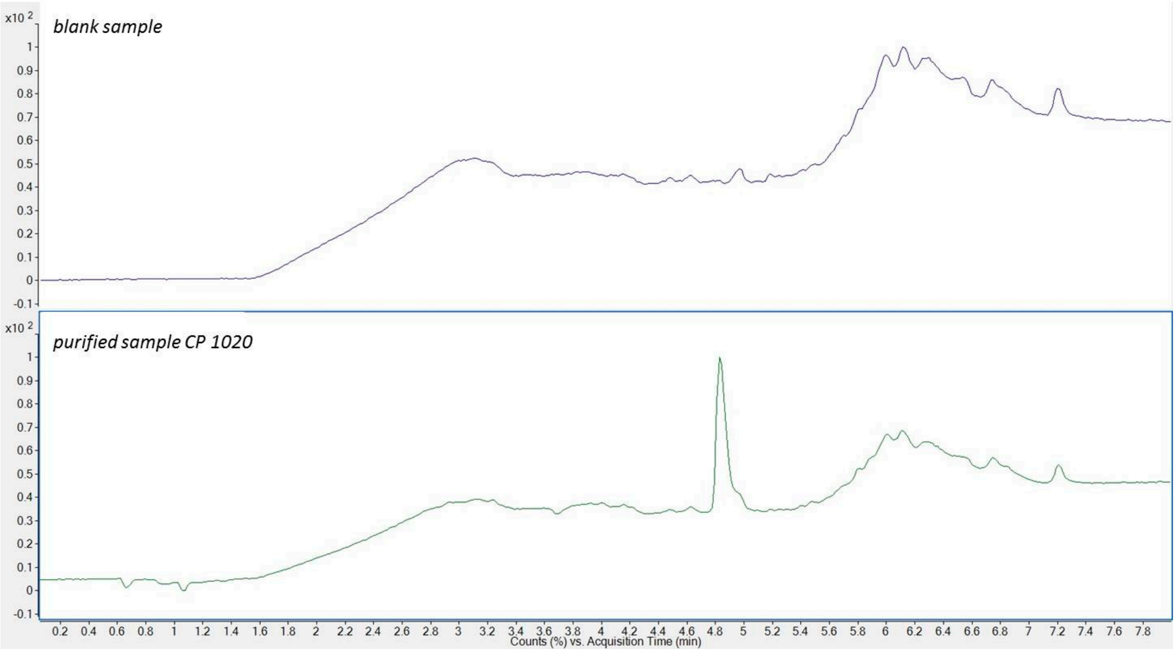

Figure S15. Total Ion Chromatogram of blank sample and purified sample CP 1020 of *Microcystis* extract after the fractionation step.

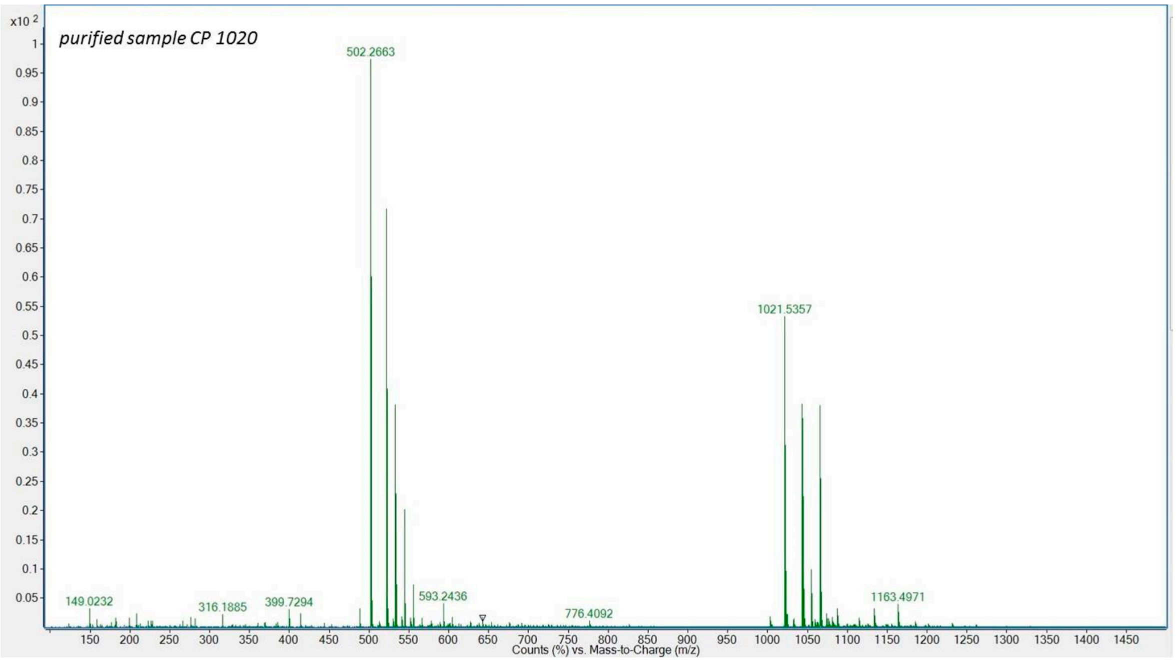

Figure S16. MS spectrum of the purified sample CP1020. Mass-to-charge 1021 is the $[M + H]^{+}$ signal and the base peak *m*/*z* 502 $[M + 2H - H_2O]^{+2}$ of the compound.

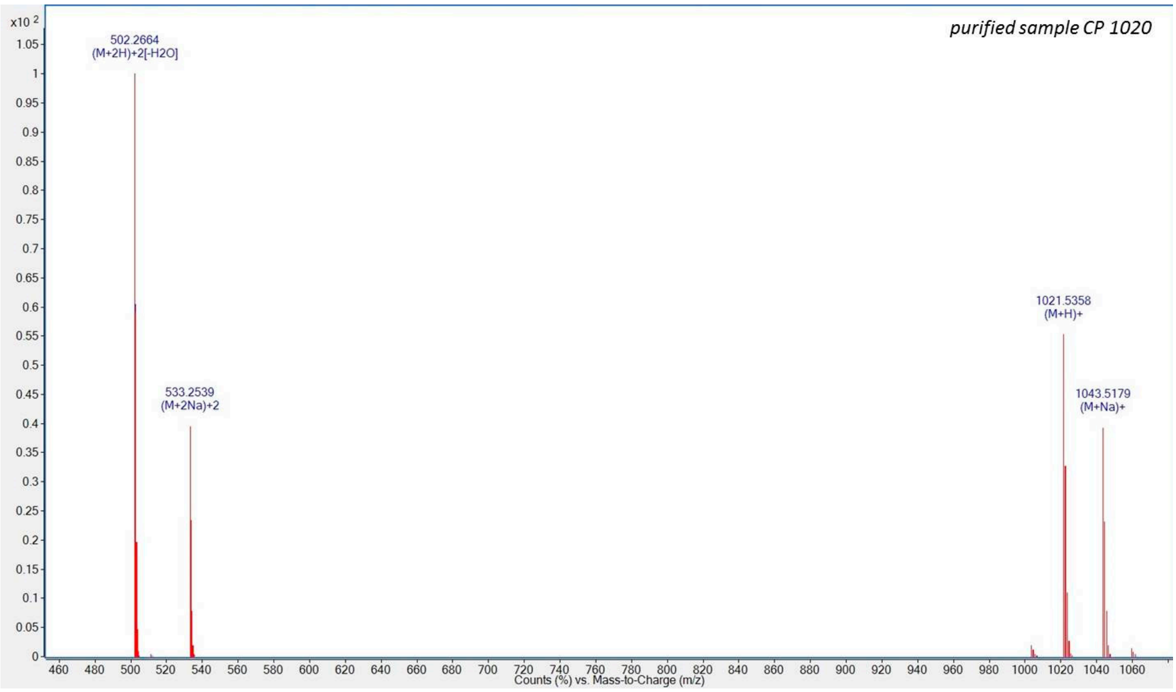

Figure 17. MS spectrum with charge carrier, neutral loss annotation and predicted isotope distribution (red bars) of CP1020.

Table S1. Characteristics of standard control sample and purified sample.

Name	Retention Time (min)	Sum Formula	Calculated Mass (Da)	Measured Mass (Da)	Mass Error (ppm)
control sample AG 828A	4.2	$C_{36}H_{53}ClN_6O_{12}S$	828.31307	828.31339	0.39
purified sample AG 828A	4.2	$C_{36}H_{53}ClN_6O_{12}S$	828.31307	828.31342	0.43
purified sample CP 1020	4.8	$C_{50}H_{72}N_{10}O_{13}S$	1020.5280	1020.5287	0.69

Primer Sequences of Primers for Transcription Analysis in Huh7 Cells and Zebrafish Eleuthero Embryos, as well as Zebrafish liver Organ Cultures Exposed to Aeruginosin 828A (Tables S2 and S3)

Table S2. Primer sequences for analysis in Huh7 cells.

Huhu 7 cells	Primer	Sequence
CYP1a	forward	AGGAGCTAGACACAGTGATTG
cytochrome P450, 1A	reverse	GTTCAGGTAGGAACTCAGATG
AHR	forward	CCAACATCACCTACGCCAGTCG
aryl hydrocarbon receptor	reverse	ACATCTTGTGGGAAAGGCAGCAG
GSTa1	forward	ATCCTCCTTCTGCCCGTATGTC
Glutathione S-Transferase a1	reverse	AAGTTCCACCAGATGAATGTCAGC
UGT1A6	forward	AGCCCAGACCCTGTGTCCTA
uridine diphospho-glucuronosyltransferase 1A6	reverse	CCACTCGTTGGGAAAAAGTCA
ODC	forward	ATGTTGCATCAGCT TTCACG
ornithin-decarboxylase	reverse	ACTCTCCCAGGCACAAGA CA
NQO	forward	CGCAGACCTTGTGATATTCCAG
NAD(P)H chinon oxidoreductase	reverse	CGTTTCTTCCATCCTTCCAGG
BIP	forward	CGA GGA GGA GGA CAA GAA GG
Binding immunoglobulin protein	reverse	GAC CTT GAA CGG CAA GAA CT
CHOP	forward	GGA GCA TCA GTC CCC CAC TT
C/EBP homologous protein	reverse	TGT GGG ATT GAG GGT CAC ATC
XBP-1s	forward	TGC TGA GTC CGC AGC AGG TG
spliced X-box binding protein 1	reverse	GCT GGC AGG CTC TGG GGA AG
GADD34	forward	CCC AGA AAC CCC TAC TCA TGA TC
growth arrest and DNA damage-inducible protein 34	reverse	GCC CAG ACA GCC AGG AAA T
HERP	forward	AAC GGC ATG TTT TGC ATC TG
homocysteine inducible ER protein with ubiquitin like domain 1	reverse	GGG GAA GAA AGG TTC CGA AG
DR5	forward	AGA CCC TTG TGC TCG TTG TC
Death receptor 5	reverse	TTG TTG GGT GAT CAG AGC AG
CJUN	forward	TCC AAG TGC CGA AAA AGG AAG
-	reverse	CGA GTT CTG AGC TTT CAA GGT
CFOS	forward	CCG GGG ATG CCT CTC TTA CT
-	reverse	CCAGGTCCGTGCAGAAGTC
CASP8	forward	CAG AGC CTG AGA GAG CGA TG
caspase 8	reverse	AGG CTG AGG CAT CTG TTT CC
IL8	forward	GAG TGC TAA AGA ACT TAG ATG TCA G
interleukin 8	reverse	GCT TTA CAA TAA TTT CTG TGT TGG C
TNFα	forward	CAG CCT CTT CTC CTT CCT GA
Tumor necrosis factor α	reverse	TGAGGTACAGACCCTCTGAT
uPA	forward	CAC GCA AGG GGA GAT GAA
Urokinase plasminogen activator	reverse	ACA GCA TTT TGG TGG TGA CTT
PAI	forward	TGC TGG TGA ATG CCC TCT ACT
Plasminogen activator inhibitor type 1	reverse	CGG TCA TTC CCA GGT TCT CTA
GAPDH	forward	GAAGGTGAAGGTCGGAGTC
Glyceraldehyde 3-phosphate dehydrogenase	reverse	GAAGATGGTGATGGGATTTC

Table S3. Primer sequences for analysis in zebrafish eleuthero-embryos and liver organ culture.

Zebrafish	Primer	Sequence
bip	forward	CGA AGA AGC CAG ATA TCG ATG
Binding immunoglobulin protein	reverse	ACG GCT CTT TTC CGT TGA AC
chop	forward	GAG TTG GAG GCG TGG TAT GA
C/EBP homologous protein	reverse	CCT TGG TGG CGA TTG GTG AA
cat	forward	AGG GCA ACT GGG ATC TTA A
Catalase	reverse	TTT ATG GGA CCA GAC CTT GG
sod	forward	GGC CAA CCG ATA GTG TTA GA
Super oxid dismutase	reverse	CCA GCG TTG CCA GTT TTT AG
c-jun	forward	ACG TGG GAC TTC TCA AAC TG
-	reverse	TCT TGG GAC ACA GAA ACT GG
dusp 5	forward	TGA AGG TCT CCA GCA TAG
dual specificty phospatase 5	reverse	GGA ATG ACG AAC TGT AGA G
p53	forward	GCT TGT CAC AGG GGT CAT TT
tumor suppressor p53	reverse	ACA AAG GTC CCA GTG GAG TG

casp3	forward	CCG CTG CCC ATC ACT A
Caspase 3	reverse	ATC CTT TCA CGA CCA TCT
bax	forward	TCA CTC GTT CAG ACC CTC AT
bcl-2-like protein	reverse	ACG CTT TCC ACG CAC AT
cry5	forward	CAT GGA GAG AAC GAA CTG GG
cryptochrome 5	reverse	GTG CAG ACA AGC AGC CGA AC
tnfα	forward	ACC AGG CCT TTT CTT CAG GT
Tumor necrosis factor α	reverse	TGC CCA GTC TGT CTC CTT CT
vtg	forward	AGC TGC TGA GAG GCT TGT TA
vitellogenin	reverse	GTC CAG GAT TTC CCT CAG T
er	forward	TGA GCA ACA AAG GAA TGG AG
estrogen receptor	reverse	GTG GGT GTA GAT GGA GGG TTT
cyp19b	forward	GGC AGT CTC TGG AGG ATG AC
cytochrome P450, 19b	reverse	CAG TGT TCT CGA AGT TCT CCA
tshβ	forward	GCA GAT CCT CAC TTC ACC TAC C
Thyroid stimulating hormone, beta	reverse	GCA CAG GTT TGG AGC ATC TCA
gst	forward	CTA TAC ATG CGG CGA AGC
Glutathione S-Transferase	reverse	CGC ATT GCT CTG GAC GAT
cyp1a	forward	CCT GGG CGG TTG TCT ATC TA
cytochrome P450, 1a	reverse	TGA GGA ATG GTG AAG GGA AG
ahr1	forward	TAG ACA GCG ATA TAC AGC AG
aryl hydrocarbon receptor 1	reverse	TCTCTCCAACACCATTCATG
ahr 2	forward	ACGGTGAAGCTCTCCCATA
aryl hydrocarbon receptor 2	reverse	AGTAGGTTTCTCTGGCCAC
nr1d1	forward	GTG AAC AAC CAG CTG CAG AA
nuclear receptor subfamily 1, group d, member 1	reverse	ACT GTA AGG CCT GGA CAT GG
per1	forward	ATG CGT GCA AGA AGT GGT G
period 1	reverse	ACG TCC TCA TTT AGC GGA CTC
Ptgds	forward	CCA TCA AGA CCA AAG GAG GA
prostaglandin D2 synthase	reverse	TCC ATT TTG TGG AAG CAT GA
esr1	forward	TGA GCA ACA AAG GAA TGG AG
ER alpha	reverse	GTG GGT GTA GAT GGA GGG TTT
abcg2a	forward	TCA TGA AGC CGG GAC TGA AC
ATP-binding cassette, sub-family G	reverse	GCT CCG TCT ATC AGC ACC TC
rpl13a	forward	AGC TCA AGA TGC CAA CAC AG
ribosomal protein L13a	reverse	AAG TTC TTC TCG TCC TCC
